# Supplementary material for: Integrated Management of the Cotton Charcoal Rot Disease Using Biological Agents and Chemical Pesticides
Source: J Fungi (Basel). 2024 Mar 26;10(4):250. doi: 10.3390/jof10040250 (PMC11050767; doi:10.3390/jof10040250)
Supplement: Supplementary file 1 [file jof-10-00250-s001.zip › jof-2819879-supplementary.pdf]

Journal of *Fungi*

MDPI

Article

Integrated Management of the Cotton Charcoal Rot Disease Using Biological Agents and Chemical Pesticides

Ofir Degani [1,2,*], Assaf Chen [2,3], Elhanan Dimant [1], Asaf Gordani [1,2], Tamir Malul [1,2] and Onn Rabinovitz [1]

[1] Plant Sciences Department, Migal—Galilee Research Institute, Tarshish 2, Kiryat Shmona 1101600, Israel; elhanand@migal.org.il (E.D.); asigordani1@gmail.com (A.G.); tamir1012011@gmail.com (T.M.); onnrab@gmail.com (O.R.)
[2] Faculty of Sciences, Tel-Hai College, Upper Galilee, Tel-Hai 1220800, Israel; assafc@migal.org.il
[3] Soil, Water and Environment Department, Migal—Galilee Research Institute, Tarshish 2, Kiryat Shmona 1101600, Israel
* Correspondence: d-ofir@migal.org.il or ofird@telhai.ac.il; Tel.: +972-54-678-0114

Supplementary Materials

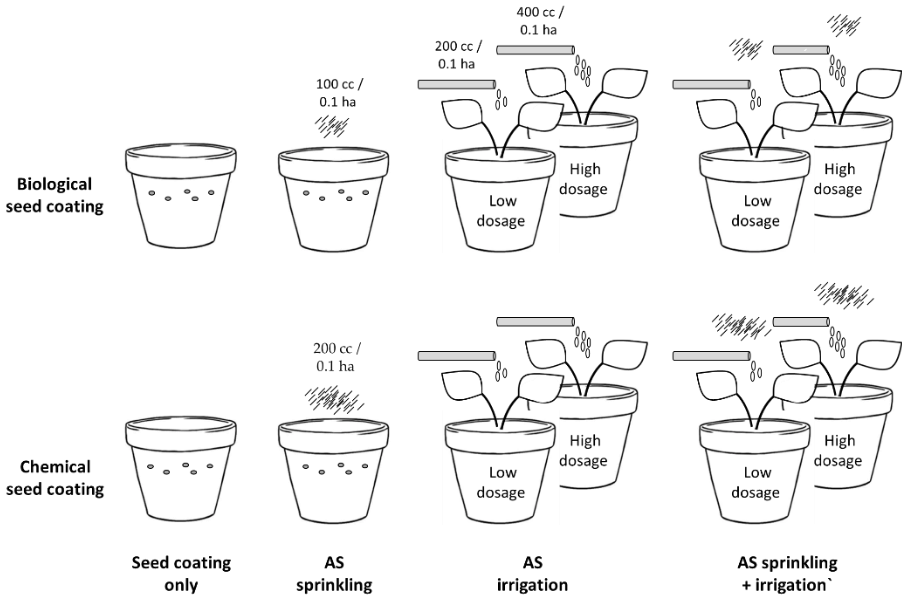

Figure S1. Experimental design of the pot assay showing the different treatments applied after sowing in substrate infected with *Macrophomina phaseolina*. Two types of seed coating were compared, biological (Trichoderma mix) or chemical (a mixture of thiram, captan, carboxin, and metalaxyl-M), solely or combined with the following five different applications of the fungicide Azoxystrobin (AS): by sprinkling at sowing, by irrigation (two dosages) on 10- and 21-day post sowing, or by sprinkling + irrigation. Healthy, non-infected control groups (which also had biological or chemical seed coating) were included. Every experimental group included seven biological replications, for a total of 98 pots.

Table S1. Meteorological data for the commercial field experiments [1].

Parameters	Value
Dates	09/04/2023–07/09/2023
Temperature (2 m above soil, °C)	24.2 ± 6.3
Humidity (%)	67.0 ± 30.8
Radiation (W/m^2)	294.4 ± 50.9
Precipitation (mm)	81.8

[1] Average data (± standard deviation) according to meteorological station Hulda 24 of The Land Conservation Division, Israel Ministry of Agriculture.

Table S2. The plants ' growth parameters and survival rate after the different treatments in the pot assay on days 29 and 52 post-sowing, expressed as a percentage of healthy, non-infected controls. [a]

		Day 29 (average / pot)						Day 52 (average / plant)				
	Treatment	Survival (%)	Leaves (no.)	Root length (cm)	Shoot height (cm)	Root weight (g)	Shoot weight (g)	Leaves (no.)	Root length (cm)	Shoot height (cm)	Root weight (g)	Shoot weight (g)
Chemical SC [b]	SC only	106%	98%	111%	95%	97%	98%	116%	102%	114%	122%	131%
	Sp	103%	102%	97%	89%	121%	101%	118%	117%	114%	138%	145%
	D200	100%	93%	90%	105%	90%	93%	107%	108%	113%	128%	118%
	D400	103%	83%	113%	91%	90%	78%	101%	79%	122%	96%	103%
	Sp + D200	91%	65%	76%	68%	72%	51%	91%	84%	95%	114%	91%
	Sp + D400	100%	84%	83%	80%	89%	70%	104%	102%	116%	113%	101%
Biological SC	SC only	97%	106%	94%	97%	94%	114%	110%	110%	110%	137%	134%
	Sp	91%	95%	88%	87%	98%	91%	107%	108%	109%	135%	133%
	D200	94%	95%	98%	92%	78%	91%	109%	120%	116%	129%	122%
	D400	94%	84%	85%	94%	73%	78%	103%	104%	118%	109%	102%
	Sp + D200	91%	85%	75%	95%	71%	85%	97%	111%	112%	100%	105%
	Sp + D400	99%	82%	83%	91%	69%	84%	106%	109%	120%	117%	112%

[a] The treatments were performed once with chemical seed coating (Chemical SC) and once with bio-coating (*Trichoderma* species mix, Biological SC). The treatments are seed coating solely (SC only), seed coating with Azoxystrobin sprinkling (Sp) or irrigation at low (D200) or high (D400) dosage, and a combination of the three (Sp + D200 and Sp + D400). The best and the least influential treatments in controlling the CRD fungus, *Macrophomina phaseolina,* are highlighted in green and pink, respectively. [b] SC – seed coating.

Table S3. *Macrophomina phaseolina* DNA in the plants' root after the different treatments in the pot assay on days 29 and 52 post-sowing, expressed as a percentage of healthy, non-infected controls. [a]

		Day 29	Day 52
Chemical SC [b]	SC only	100%	100%
	Sp	38%	49%
	D200	39%	23%
	D400	116%	25%
	Sp + D200	144%	127%
	Sp + D400	280%	66%
Biological SC	SC only	81%	55%
	Sp	49%	35%
	D200	42%	22%
	D400	49%	160%
	Sp + D200	107%	111%
	Sp + D400	163%	263%

[a] The treatments were performed once with chemical seed coating (Chemical SC) and once with bio-coating (*Trichoderma* species mix, Biological SC). The treatments are seed coating solely (SC only), seed coating with Azoxystrobin sprinkling (Sp) or irrigation at low (D200) or high (D400) dosage, and a combination of the three (Sp + D200 and Sp + D400). The best and the least influential treatments in controlling the CRD fungus, *M. phaseolina*, are highlighted in green and pink, respectively. [b] SC – seed coating.

Table S4. Assessment of the yield, symptoms, and *Macrophomina phaseolina* DNA in the plants' roots after the different treatments in the field trial, expressed as a percentage of the control—chemically (Captan) coated seeds plants. [a.]

		Yield	Symptoms	qPCR	
	**Treatment**	**Day 210**	**Day 151**	**Day 74**	**Day 151**
**Chemical SC [b]**	**SC only**	100%	100%	100%	100%
	**Sp**	105%	116%	166%	84%
	**D200**	101%	156%	140%	115%
	**D400**	113%	115%	142%	63%
	**Sp + D200**	101%	148%	131%	123%
	**Sp + D400**	109%	94%	117%	216%
**Biological SC**	**SC only**	117%	94%	128%	78%
	**Sp**	102%	73%	99%	93%
	**D200**	104%	127%	122%	97%
	**D400**	116%	94%	207%	201%
	**Sp + D200**	98%	126%	135%	127%
	**Sp + D400**	112%	99%	83%	63%

[a] The treatments were performed once with chemical seed coating (Chemical SC) and once with bio-coating (*Trichoderma* species mix, Biological SC). The treatments are seed coating solely (SC only), seed coating with Azoxystrobin sprinkling (Sp) or irrigation at low (D200) or high (D400) dosage, and a combination of the three (Sp + D200 and Sp + D400). The best and the least influential treatments in controlling the CRD fungus, *M. phaseolina*, are highlighted in green and pink, respectively. [b] SC – seed coating.
